# Substoichiometrically Different Mitotypes Coexist in Mitochondrial Genomes of *Brassica napus* L

**DOI:** 10.1371/journal.pone.0017662

**Published:** 2011-03-10

**Authors:** Jianmei Chen, Rongzhan Guan, Shengxin Chang, Tongqing Du, Hongsheng Zhang, Han Xing

**Affiliations:** State Key Laboratory of Crop Genetics and Germplasm Enhancement, Nanjing Agricultural University, Nanjing, People's Republic of China; University of Georgia, United States of America

## Abstract

Cytoplasmic male sterility (CMS) has been identified in numerous plant species. *Brassica napus* CMS plants, such as Polima (pol), MI, and Shaan 2A, have been identified independently by different researchers with different materials in conventional breeding processes. How this kind of CMS emerges is unclear. Here, we report the mitochondrial genome sequence of the prevalent mitotype in the most widely used pol-CMS line, which has a length of 223,412 bp and encodes 34 proteins, 3 ribosomal RNAs, and 18 tRNAs, including two near identical copies of *trnH*. Of these 55 genes, 48 were found to be identical to their equivalents in the “nap” cytoplasm. The *nap* mitotype carries only one copy of *trnH*, and the sequences of five of the six remaining genes are highly similar to their equivalents in the *pol* mitotype. Forty-four open reading frames (ORFs) with unknown function were detected, including two unique to the *pol* mitotype (*orf122* and *orf132*). At least five rearrangement events are required to account for the structural differences between the *pol* and *nap* sequences. The CMS-related *orf22*4 neighboring region (∼5 kb) rearranged twice. PCR profiling based on mitotype-specific primer pairs showed that both mitotypes are present in *B. napus* cultivars. Quantitative PCR showed that the pol cytoplasm consists mainly of the *pol* mitotype, and the *nap* mitotype is the main genome of nap cytoplasm. Large variation in the copy number ratio of mitotypes was found, even among cultivars sharing the same cytoplasm. The coexistence of mitochondrial mitotypes and substoichiometric shifting can explain the emergence of CMS in *B. napus*.

## Introduction

The major contribution of the mitochondrion to plant growth and development is the production of energy [1]. In some higher plants, the economic significance of mitochondria is boosted by its role in the determination of cytoplasmic male sterility (CMS). To outline characteristics of the plant mitochondrial genome and identify CMS-related genes, the mitochondrial genomes of more than 10 fertile or sterile plant species have been sequenced, including *Arabidopsis thaliana* [2], *Beta vulgaris* [3], [4], *Oryza sativa* [5], [6], [7], *Brassica napus* [8], *Zea mays* [9], [10], *Nicotiana tabacum* [11], *Triticum aestivum* [12], *Vitis vinifera* [13], *Citrullus lanatus,* and *Cucurbita pepo* [14]. Research revealed that unique deleterious genes inserted into the mitochondrial genome confer CMS traits on natural plant germplasm [15], [16].

In higher plants, natural CMS has been identified in more than 150 species over the past century [17]. CMS emergence undoubtedly stems from alteration of the mitochondrial genome [15], [17], but the question is whether this kind of genomic alteration is the result of toxic genes being inserted during the past century or from the substoichiometric shifting of mitochondrial DNA molecules. Research in a few crops has demonstrated that substoichiometric shifting results in the inherent CMS-triggering mitotype dominating mitochondrial genome constituents [18]–[20], which results in the occurrence of CMS. In the common bean, a progenitor mitochondrial form containing a three-molecule CMS-associated configuration is universally present in the germplasm [18], and substoichiometric shifting can explain the emergence of CMS. In cybrids between CMS radish and *B. napus*, mitochondrial genomic components of the two species coexist in stable rapeseed progenies, and the phenomena of substoichiometric shifting has been observed between male fertile and sterile plants [21].

In *B. napus,* at least five natural occurrences of CMS have been independently reported [22]–[27]. Genetic experiments and restriction mapping have shown that the CMS lines can be classified as two cytoplasmic groups, one including T-CMS and S-CMS [22]–[24], which is commonly referred to as the “nap” cytoplasm present in normal fertile accessions, and the other group as “pol” cytoplasm including pol-CMS, MI CMS, and shaan 2A CMS [25]–[27], which has been the most widely applied line in the world. During the past few decades, the fact that the grouped CMS accessions from multiple independent findings have the same genetic regulation has been a perplexing issue for researchers [28]. In order to identify the mechanism for natural CMS in *B. napus,* the main mitochondrial genome of pol-CMS was sequenced and compared to the sequence of Westar (nap cytoplasm), uncovering structural and evolutionary differences. Based on the mitotype-specific sequences of the mitochondrial genome in *B. napus*, the constituents of the mitochondrial genome were detected by PCR amplification.

## Results

### The *pol* mitochondrial genome

A single circular mitochondrial genome 223,412 bp (EMBL accession number: FR715249) in size was obtained by shotgun sequencing. Because of heteroplasmy in *B. napus*, we refer to the mtDNA sequence of the fertile rapeseed variety Westar as the *nap* mitotype, in contrast to the polima-derived *pol* mitotype. The *pol* mitotype sequence is 1,559 bp longer than that of the *nap* mitotype, and the G+C content of the two mitotypes is 45.22% and 45.19%, respectively. Sequence alignment showed that 3.63% of *nap* and 4.53% of *pol* is mitotype-specific.

**Figure 1:**
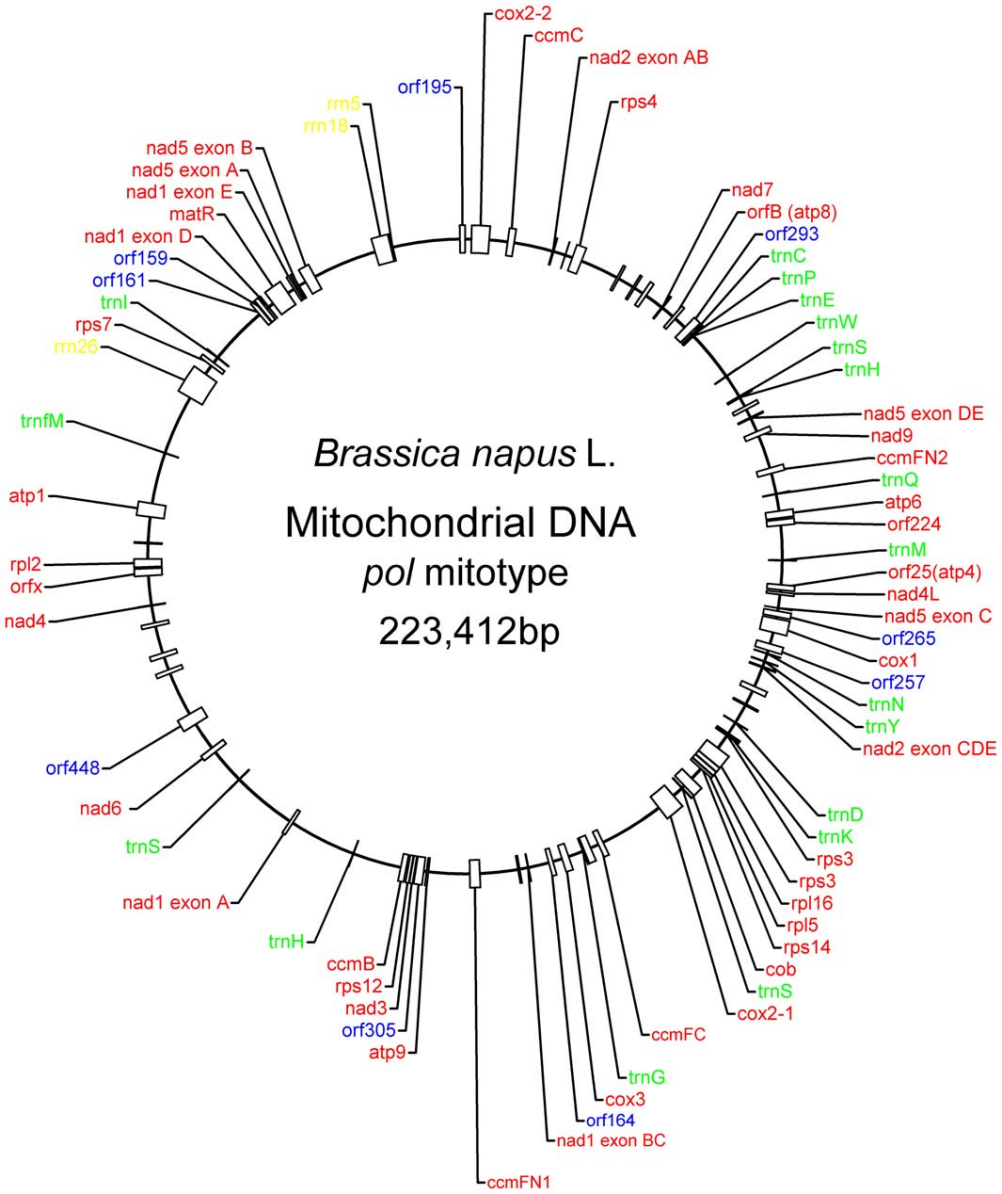
Organization of the *pol* mitotype in *B. napus*. A total of 55 genes with known function were identified, including 34 protein-coding genes (red), 3 ribosomal RNA genes (yellow), and 18 tRNA genes (green). Nine (out of 44) putative ORFs encoding at least 150 amino acids are marked in blue.

The *pol* sequence encodes 34 proteins, three ribosomal RNAs, and 18 tRNAs, constituting 17.34% of the genome (Figure 1). The sequence includes two near identical copies of *trnH* that differ by only one base pair, whereas only a single copy is present in the *nap* mitotype. Of the 55 genes present in *pol*, 48 have an identical copy in *nap*, implying that the known mitochondrial genes are well conserved in *B. napus*. Five of the six remaining genes in *nap* differ only marginally from their counterparts in *pol* (the exception was *orf224*). The *atp1* sequences differ by one synonymous single nucleotide polymorphism (SNP), as do the *trnC* and *trnE* sequences. One non-synonymous SNP in *cox1* produces a proline to leucine switch, but this has no effect on gene function. Similarly, a single SNP produces a proline to serine switch in *cox2*. The alignment of the *pol* and *nap* versions of *orf224*, which is generally accepted as the determinant of CMS in *pol*, revealed 84% nucleotide similarity and 77% peptide similarity.

We found that the *pol* mitotype comprises 44 putative open reading frames (ORFs) encoding at least 100 amino acid residues. Of these ORFs, 35 are identical to their equivalents in *nap*, but the other ORFs are different (Table S1). *Orf122* and *orf132* are both present in *pol* but not in *nap*, whereas *orf117b* is unique in *nap*. None of these three sequences aligned with any known sequence in GenBank. Thus, the sequences were inferred to be the result of large insert sequences in the mitochondrial genome. *Orf261* in *nap* and *orf265 in pol* partially aligned and were inferred to be generated by the rearrangement of syntenic regions in the mitochondrial genome.

### Reorganization of the mitochondrial genome

**Figure 2:**
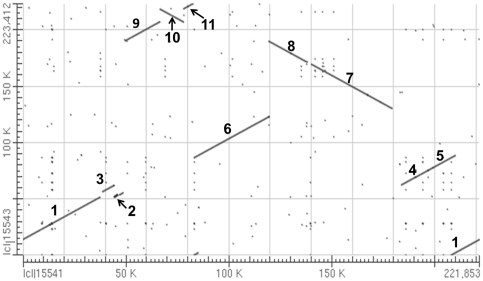
Alignment of the *nap* and *pol* mitotype genomes. The numbers refer to the syntenic regions with the *pol* mitotype sequence as a reference. Comparisons between *nap* (horizontal axis) and *pol* (vertical axis) indicated that the nucleotide sequences of the syntenic region are highly conserved, but the syntenic orders and directions were evolutionally rearranged.

Analysis of the syntenic regions of *pol* and *nap* using the bl2seq algorithm suggested that the sequence as a whole consists of 11 syntenic regions (similarity >99%, length >2 kb). The orientation of eight of the regions is identical, but in the other three it is reversed (Figure 2). The syntenic regions are largely discrepant in the two genomic positions, though the genetic sequences are well conserved (Figure 2). A minimum of five recombination events is required to account for the structural differences between the two mitotypes.

**Figure 3:**
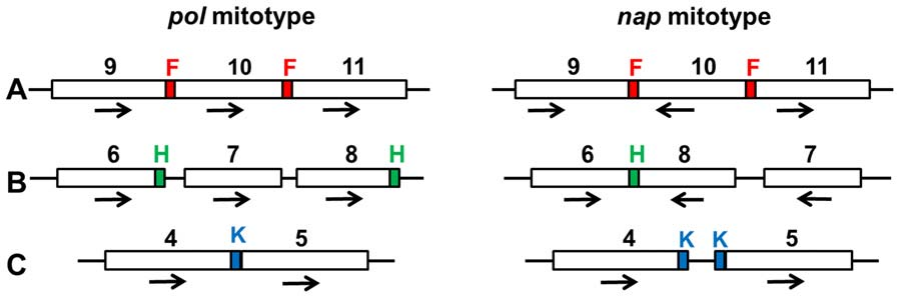
Syntenic reordering and structural genomic changes caused by short repetitive sequences. The numbers refer to the syntenic regions as in Figure 2. The orientation of the sequence is shown by an arrow. (**A**) A pair of inverted repeats (in red, F) may have induced the re-orientation of region 10. (**B**) In *pol*, a pair of inverted repeats (in green, H) is located at the end of linked regions 8 and 7, but in *nap*, this region is re-orientated and contains a small deletion of a sequence of unknown origin and fewer repeats due to an overlap of H at the terminus of region 6. (**C**) A 50 bp sequence (in blue, K) is located between syntenic regions 4 and 5 in *pol*, whereas two copies of K are present at this location in *nap*, accompanied by a 44 nucleotide insertion of unknown origin.

Repeated sequences in the *pol* mitotype were analyzed. A pair of large repeats 2427 bp in size identified in *pol* are also found in *nap* [8] and related to the formation of the multipartite structure of the *Brassica* mitochondrial genome, including one master circle and two smaller subgenomic circles through homologous recombination [29], [30]. The *pol* sequence also contains multiple copies of various 30–500 bp repeats; applying a 90% sequence similarity criterion to define a repeat, these sequences account for 4.75% of the genome and are arranged in both direct and inverse orientation. The repeats probably provide the opportunity for mtDNA sequence evolution as illustrated in Figure 3. The pair of inverted 146 bp repeats (F) on either side of syntenic region 10 may account for the changed orientation of this region in the two mitotypes (Figure 3A). A pair of inverted 233 bp repeats (H) is present at the end of syntenic regions 8 and 6 in *pol,* whereas the whole of regions 8 and 7 is inverted in *nap* following rearrangement, accompanied by the deletion of one short repeat (Figure 3B). Short repeats seem to be associated with inserted or deleted sequences of mtDNA because the 50 bp sequence (K) between syntenic regions 4 and 5 in *pol* is present as two copies in the *nap* mitotype, along with the insertion of an anonymous 44 bp sequence (Figure 3C). Short repeats providing cues to the genome reorganization may correspond to recombination hotspots in plant mitochondrial genomes [31].

**Figure 4:**
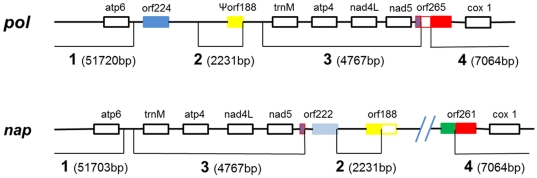
Structural polymorphism in the region surrounding *orf224*. The numbers refer to the syntenic regions as in Figure 2. At least two recombination events are inferred in this region. One of these, located within *orf265* in *pol*, splits this sequence into three segments, and in nap the terminus of *orf265* generates *orf261* by fusing to a neighboring sequence; the second event occurs between regions 2 and 3 resulting in the formation of *orf188,* a combination of the terminal 90 bp of region 2 and a new neighboring sequence in the *nap* mitotype.

A more detailed analysis of the *orf224* region, which is reported to be the CMS-associated region for *pol* [32], was carried out. The *pol* and *nap* mitotypes differed by at least two recombination events (Figure 4). The first recombination event, within *orf265* of pol mitotype, broke the region into three parts. In the *nap* mitotype, the frontal 78 bp and terminal 467 bp of the *orf265* sequence were separated, together with regions 3 and 4, respectively, whereas a 253 bp segment in the middle of the ORF was lost in the course of the rearrangement. The 467 bp *orf265* sequence was then attached to a neighboring sequence, generating the *nap* form of *orf261.* The second recombination event occurred at the end of region 2. Another putative gene, *orf188,* in the *nap* mitotype appeared because of the relocation of region 2. Only 90 bp of the *nap orf188* sequence remained in *pol*, probably due to evolutionary events that resulted in a sequence loss. Summarily, this region is more frequently rearranged than other regions in genomic sequences.

### Substoichiometrically different *pol* and *nap* mitotypes coexist in *B. napus*

**Figure 5:**
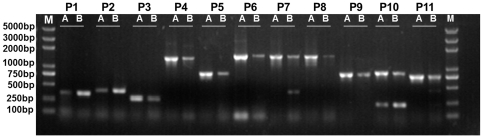
PCR amplification of the pol-CMS line and its maintainer. The *pol* and *nap* mitotypes are both present in pol-CMS line NH3A (**A**) and its maintainer NH3B (**B**), but are substoichiometrically differentiated in these cytotypes. Primer pairs P1 and P2 targeted the *nap* mitotype-specific sequences *orf117b* and *orf222*, respectively, whereas P3-9 and P11 targeted *pol* mitotype-specific sequences. P10 amplified two distinct fragments 841 bp (*pol*-specific) and 226 bp (*nap*-specific) in size. All PCR reactions used the 10 ng of mtDNA template. All primer pairs were able to amplify target fragments in both lines, but amplification differed in the two lines. The PCR assay indicated that the *nap* and *pol* mitotypes coexisted in rapeseed, but the content was substoichiometrically different in the two cytoplasm types. M: DNA ladder.

In order to demonstrate our inference about the coexistence of *pol* and *nap* mitotypes in *B. napus* cytoplasm, we designed 11 pairs of mitotype-specific PCR primers according to the two entire mitotype sequences. Using mtDNA extracted from pol-CMS line NH3A and its nap cytoplasm maintainer line NH3B as templates, PCR amplification showed that all primers amplified fragments regardless of the line (Figure 5). The profile generated by the multiple-primer P10 consisted of two bands of contrasting intensity(Figure 5). The results indicated that the mitochondrial population in both cytotypes was mixed. In addition, 14 materials with different origins were used in the PCR reactions. PCR with the P1 (*nap*-specific) and P5 (*pol*-specific) primers indicated that both primers were effective at amplifying all of the templates (Figure S1 and S2), demonstrating the coexistence of the two mitotypes. Sequencing of the various amplicons generated from the 11 primer pairs confirmed that all PCRs amplified their target sequences. The PCR profiles successfully established the coexistence of the two mitotypes within one *B. napus* plant. However, these results do not mean that the main genomes in the cytoplasm of different genetic lines are the same. Under identical PCR conditions with the same controlled mtDNA template concentration, the amplification was greater for the pol-CMS line than its maintainer when *pol* mitotype-specific primers were used (Figure 5). On the other hand, when *nap* mitotype-specific primers were used, the amplification was greater in the maintainer line compared to the CMS line. These results indicated that differences exist at the substoichiometric level of mtDNA molecules between the pol-CMS line and its maintainer. The main genome in the pol-CMS plant is the *pol* mitotype, whereas the main genome in the nap cytoplasm is *nap*. In other words, the cytoplasmic difference may be explained by the substoichiometric complexity of the rapeseed mitochondrial genome, such as that observed in the common bean [18], [19]. These interesting and surprising truths upset the conceptual framework of the rapeseed mitochondrial genome in regards to the pol and nap cytotypes.

### *Orf222* to *orf224* copy number ratio in *B. napus*

The ratio of *orf224* and *orf222* copy numbers, which are popularly accepted as CMS-related genes, was estimated using a TaqMan qPCR platform. The results indicated that the ratio was cytoplasm-dependent. In pol-CMS lines, the *orf224* copy number was greater than that of *orf222*, but the predominant gene was *orf222* in maintainer lines and other male fertile cultivars carrying *nap* cytoplasm (Table 1). These quantifying results are consistent with the PCR assays mentioned above. Obviously, some substoichiometric shifting of *orf224/orf222* occurred between pol-CMS lines and their maintainers with *nap* cytoplasm. Substoichiometric shifting from *orf222* to *orf224* (or from *orf224* to *orf222*) in different materials represents a cytoplasmic difference, and the substoichiometric shifting appears to be the determinant of the male fertility/sterility phenotype in *B. napus*.

**Table 1:** Copy number ratio of *orf222* and *orf224* in different accessions of *B. napus.*

*pol* cytoplasm	*orf224/orf222*	*nap* cytoplasm	*orf222/orf224*
NH3A	15.00	NH3B	1097.71
NH12A	1929.68	NH12B	7479.48
NH15A	287.25	NH15B	15771.91
NH18A	88.48	NH18B	1630.26
NH21A	15652.38	NH21B	4401.30
NH923A	18.39	NH923B	445.26
		Westar	11337.91
		Tapidor	36.17
		Huaiyin 16	155209.38
		Zheshuang 72	135117.61
		Westar in 2010	164059.11*
		Tapidor in 2010	746.43*

* repeated determination in 2010. The rest were determined in 2009.

However, we also noted variation in the copy number ratio among cultivars sharing the same cytotype. In cultivars carrying *pol* cytoplasm, the *orf224*:*orf222* ratio ranged from 15 to >15,000; in cultivars with *nap* cytoplasm, the *orf222*:*orf224* ratio ranged from 36 to >150,000 (Table 1). The abundance of sublimons (i.e. alternative genomes) was roughly two to six orders of magnitude less than that of the prevalent mitotype. This dramatic variation in the substoichiometric ratio among accessions sharing the same cytoplasm may be under nuclear control. The *orf222*:*orf224* copy number ratio in the Westar cultivar was consistently higher than that of the Tapidor cultivar, though the ratio was not stable from year to year (Table 1), presumably as a result of an interaction with the environment.

### Sequence evolution in *B. napus*

**Figure 6:**
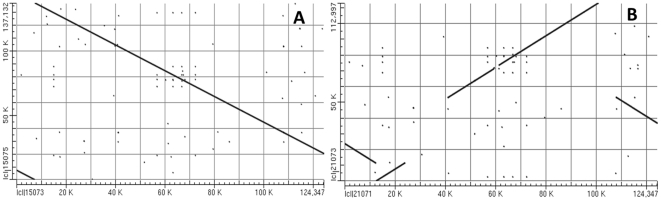
Alignment of the *B. rapa* mitochondrial segment with the two *B. napus* mitotype sequences. The 124 kb *B. rapa* mitochondrial segment was plotted on the horizontal axis against the *pol* subgenome (**A**) or *nap* subgenome (**B**) on the vertical axis.

A sequence comparison of the *pol* and *nap* mitotypes identified 197 SNPs (102 transitions, 95 transversions), equivalent to a polymorphism rate of 8.9 bp per 10,000 bases. As many as 104 of the SNPs were located within the *orf224*/*orf222* genes. Only six SNPs were identified in other genes of known function, indicating a much higher level of sequence conservation. Although reconstructing the evolution of the *pol* and *nap* mitotypes is not possible, the former is likely more primitive. The evidence for this conclusion is that the structure of the *pol* mitotype is closely related to that of a 124 kb *Brassica rapa* mitochondrial BAC clone (GenBank accession number AC172860.1) that is the likely ancestor of *B. napus* (Figure 6). Another reason is that restorer in the natural germplasm population is sparse for the pol-CMS line, but popular for the nap-CMS line, implying that *nap* cytoplasm is more accommodated evolutionarily than *pol* cytoplasm.

## Discussion

The coexistence of mitotypes has been discovered by only a few gene detection experiments in wheat [33], maize [34], pearl millet [20], and cybrids [21], and is probably common in higher plants. In the common bean, coexisting mitochondrial genome molecules have been systematically demonstrated through restriction mapping. We showed here for the first time using a combination of genome sequencing and PCR assays that the *pol* and *nap* mitotypes coexist within one *B. napus* plant.

The mitochondrial genome in higher plants is very stable, maintaining plant survival in natural environments to achieve its important functions in living activities, so it is not probable that so many cases of CMS emerge only by evolution of the mitochondrial genome over one century. Substoichiometric shifting of coexisting mitotypes in plants may be an explanation of the phenomena related to CMS findings. The mitotype diversification cannot be explained by evolutionary events occurring over a short time, and the mitotypes are probably evolutionary products of a long time-span and coexisting in the mitochondrial population. The presence of mitochondria carrying alternative, rearranged genomes (sometimes referred to as “sublimons”) has been associated with phenotypic switching, tissue differentiation, cytotype evolution, and various nuclear-cytoplasmic interactions in higher plants [35]. The means by which sublimons are maintained, propagated, and transmitted largely remain unexplored. However, the sublimons do appear to be stable and, thus, able to affect pollen fertility via substoichiometric shifting.

We provided evidence that the *B. napus* mitochondrial genome includes at least two distinct mitotypes that are present in variable ratios in both CMS and male fertile cultivars. *B. napus* cultivars sharing the same cytotype can vary considerably with respect to the copy number ratio; therefore, the value of the copy number ratio is controlled, at least to some extent, by the nuclear genotype. In the common bean, a dominant allele in a nuclear gene can act to limit the copy number of mtDNA containing the CMS-associated *pvs-orf239* sequence. Environmental factors also exert an influence on this ratio [18]. Both the nap and pol CMS trait was selected during the course of conventional breeding, apparently because in a particular confluence of nuclear genotype and environment, the copy number of the critical sublimon amplified sufficiently to exert a phenotypic effect (Figure S3). Our results, as well as previous conclusions in plant species [19], show that the conditions for the substoichiometric shifting of mitotypes are natural and common. Generally, when the alternative mitotype with malicious genes is accompanied by particular nuclear genotypes, the plants may manifest CMS phenotypes [18]. The model for the natural emergence of pol-CMS is shown in Figure S3 and probably applies to other plants.

**Figure 7:**
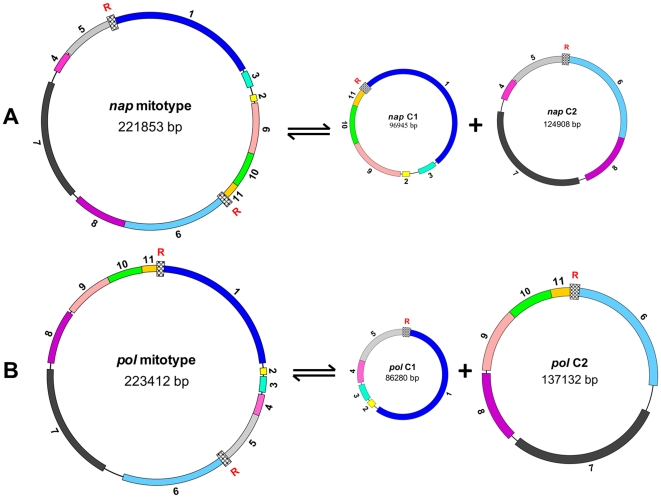
The tripartite mitochondrial genomic structure of the mitotypes. The numbers refer to the syntenic regions as in Figure 2. Highly or completely homologous regions are indicated by color. (**A**) *nap;* (**B**) *pol*. R: 2.4 kb repetitive sequence.

In higher plants, mtDNA molecules exist in many forms [35], but a multiple partite structure may still not be denied [36]. Based on the two 2.4 kb sequence repeats in the mitotypes, the *pol* mitotype is inferred as containing one master circle accompanied by two smaller circles 86.2 kb and 137.1 kb in size (Figure 7B), which is different from the *nap* mitotype (Figure 7A). Assuming that the two mitotypes are present in an equimolar amount in an oilseed rape cell, the mitochondrial genome as a whole can be expected to form a six-circle structure comprising two master and four smaller circles.

A major difference between *nap* and *pol* mitotypes is localized sequence rearrangement. The short repeated sequences in higher plant mitochondria are usually inactive and play a central role in irreversible recombination to produce a new stable mitochondrial genome structure [36]. The *pol* mitotype also contains numerous short repeated sequences, including direct repeats and inverted repeats; those located at the edge of rearranged syntenic regions may represent the vestiges of past recombination events that restructured the genome. *Nap* and *pol* mitotypes also have significant differences at the nucleotide level. In the pol-CMS of *B. napus*, expression analysis has shown that the co-transcription of *orf224* and *atp6* alters the expression of *atp6*, which is associated with CMS [32], [37]. In addition to the sequence polymorphism within *orf224*, both *orf118* and *orf265* differ between *pol* and *nap*, whereas *orf122* and *orf132* are *pol*-specific. Whether the *pol*-specific ORFs play any determining role in CMS is still unknown.

## Materials and Methods

### Plant material

Six pol-CMS lines, their corresponding maintainers, and four commercial cultivars were analyzed for mitotype constitution and copy number: NH3A/B (CMS line/maintainer), NH12A/B, NH15A/B, NH21A/B, NH18A/B, and NH923A/B (bred by the present authors). The four commercial cultivars were Westar (from Canada), Tapidor (from France), and Huaiyin 16 and Zheshuang 72 (from China). The source of mitochondrial DNA for genome sequencing was pol-CMS line NH12A. These plants were planted in Jiangpu experimental station of Nanjing Agricultural University in the Autumn of 2008, harvested in 2009, and mitotype copy number determined in the Summer of 2009 with their abundant seeds. The determinations for Westar and Tapidor were repeated in 2010.

### Isolation of mtDNA

The mtDNA was purified from 7-day-old etiolated seedlings germinated on an artificial medium in the dark at 25°C. The DNA isolation method was a modification of the method used in tobacco [38]. All of the extraction steps were performed at 4°C unless stated otherwise. A 250 g sample of seedling material was homogenized in 1000 ml buffer (0.5 M sucrose, 50 mM Tris-HCl, 10 mM EDTA, 1% bovine serum albumin, 5 mmol/L *β*-mercaptoethanol, 1.5% polyvinyl-pyrrolidone, pH 7.5). The homogenate was filtered through a layer of cheesecloth and two layers of miracloth, and centrifuged at 3000 g for 15 min. The resulting supernatant was then centrifuged at 17,000 g for 15 min and the pellet re-suspended in buffer A (0.5 M sucrose, 50 mM Tris-HCl, 10 mM MgCl_2_, and 25 µg/ml DNase (Roche 104159), pH 7.5) at a 5∶1 (g/ml) ratio and held at room temperature for 1 h. The suspension was then centrifuged at 18,000 g for 20 min and the pellet re-suspended in 30 ml buffer B (0.6 M sucrose, 10 mM Tris-HCl, 20 mM EDTA, pH 7.5). The suspension was centrifuged once more at 18,000 g for 20 min. Finally, the pellet was re-suspended in the same buffer and layered onto a step gradient consisting of 1.45 M and 1.2 M sucrose solution containing 10 mM Tris-HCl and 20 mM EDTA (pH 7.5). The gradient was centrifuged at 72,000 g for 90 min. Purified mitochondria were removed from the 1.45 M-1.2 M interphase, diluted with two volumes of buffer C (10 mM Tris-HCl, 20 mM EDTA, pH 7.5), and centrifuged at 18,000 g for 20 min. The final pellet was gently re-suspended in 10 ml lysis buffer (50 mM Tris–HCl, 10 mM EDTA, 1% SDS, and 200 µg/ml proteinase K(Sigma)) and incubated at 37°C for 3 h. Ammonium acetate was added to reach a concentration of 0.8 M, and the preparation was extracted twice in phenol/chloroform. The mtDNA was precipitated overnight in 2.5 volumes of 100% ethanol and centrifuged at 18,000 g for 20 min. The pellet was washed twice with 70% ethanol, and then air-dried and re-suspended in sterile distilled water.

### Sequencing strategy

The mtDNA was sonicated randomly and size-fractionated by agarose gel electrophoresis. The 1.6–4 kb fraction was cloned into the pUC19 vector. The inserts were subjected to cycle sequencing (BigDye terminator v3.1 Cycle Sequencing kit, Applied Biosystems, USA). A set of 2,592 reads was obtained, which is equivalent to 11x coverage of the *B. napus* mitochondrial genome. The individual reads were assembled into five contigs using Phred-Phrap software (http://www.phrap.org/). Gaps between the contigs were filled by PCR.

### Detection of coexisting mitotypes

A set of 11 PCR primer pairs (Table S2) was based on the *pol* and *nap* mitotype sequences, of which eight (P3-9, P11) are *pol*-specific. P1 targeted *orf117b* and P2 *orf222*, both of which are *nap*-specific. P10 was a multiple-primer designed to amplify both *pol* and *nap* mitotype templates, specifically generating an 841 bp *pol*-specific and 226 bp *nap*-specific amplicon.

The mtDNA samples were amplified using the primers in 25 µl PCR reactions containing 50 µM dNTPs, 0.2 µM of each primer, 1 µl template, 2.5 µl Taq polymerase buffer, and 1 U Taq polymerase (TIANGEN, China). The PCR was programmed as follows: an initial step of 94°C/5 min, then 30 cycles of 94°C/30 s, 53°C–60°C/30 s, and 72°C/90 s, followed by a final amplification step of 72°C/10 min. All PCR products were sequenced directly.

### Determination of copy number ratios

A quantitative PCR platform (TaqMan) was used to estimate the copy number of specific mitotypes. The sequences of the relevant primers and TaqMan probe were designed from the *orf224* and *orf222* sequences, which were specific for the *pol* and *nap* mitotypes, respectively (Table S3). The probe was 5′-labeled with FAM and 3′-labeled with TAMRA. All primers and probes were obtained from Invitrogen Biotechnology (China). Each 20 µl PCR contained 10 µl premix EX Taq (2x) (TaKaRa), 0.3 µM of each primer, 0.2 µM TaqMan probe, 0.4 µl Rox reference dye II, and 5 µl template. The two step PCR was carried out on an ABI PRISM 7500 Fast sequence detection system (Applied Biosystems) using the following cycling program: 95°C/50 s, then 40 cycles of 95°C/3 s, 58.5°C/30 s. The analysis of each mtDNA sample was performed in triplicate. In order to check the amplification efficiency (E) of each primer pair/probe, validation experiments were performed using a 10-fold serial dilution of mtDNA. The E of each primer pair/probe was 99.7% and 100.1%, respectively. The copy number ratio of *orf224* and *orf222* was determined from the *Ct* values and E.

### Sequence analysis

The NCBI database was searched using the blast network service and bl2seq (http://www.ncbi.nlm.nih.gov/). A tRNA gene search was carried out using the tRNA scan-SE service (http://www.genetics.wustl.edu/eddy/tRNAscan-SE/). Small dispersed repeats were defined as sequences of identical length (30–500 bp) that were found more than once in the genome and at least 90% identical. SNPs were identified by applying default settings in Mauve software [39]. Peptide and nucleotide sequences were aligned by ClustalX 1.81 using default settings [40].

## Supporting Information

Figure S1 **PCR analysis of a panel of male fertile and pol-CMS cultivars using P1**. Analysis was based on the nap-specific primer pair P1, which targeted *orf117b*, but amplification occurred in both nap- and pol-CMS lines. Huaiy16: Huaiyin16; Zhesh72: Zheshuang72; M: DNA ladder.
(TIF)

Figure S2 **PCR analysis of a panel of male fertile and pol-CMS cultivars using P5.** Analysis was based on the pol-specific primer pair P5. Amplification of the target fragment occurred in both nap- and pol-CMS lines. Huaiy16: Huaiyin16; Zhesh72: Zheshuang72; M: DNA ladder.
(TIF)

Figure S3 **The coexistence and stoichiometric shifting of the** ***nap*** **and** ***pol*** **mitotypes in** ***B. napus.*** *Nap* and *pol* mitotypes coexisted in *B. napus*, but the relative content of the two mitotypes was different in the two cytoplasm types. In the nap cytotype, *nap* is the prevalent mitotype and *pol* is present as a sublimon. In the pol cytotype, *pol* is the prevalent mitotype and *nap* is present as a sublimon. Under certain conditions, including nuclear genotypes and environmental stress, sublimons may be amplified and accumulate to take over the role of the main genome.
(TIF)

Table S1 **Different ORFs between** ***nap*** **and** ***pol*** **mitotypes.**
(DOC)

Table S2 **PCR primer sequences.**
(DOC)

Table S3 **Primers and probes for TaqMan qPCR.**
(DOC)
