# Supplementary figures and images for: Substoichiometrically Different Mitotypes Coexist in Mitochondrial Genomes of Brassica napus L
Source: PLoS One. 2011 Mar 10;6(3):e17662. doi: 10.1371/journal.pone.0017662 (PMC3053379; doi:10.1371/journal.pone.0017662)

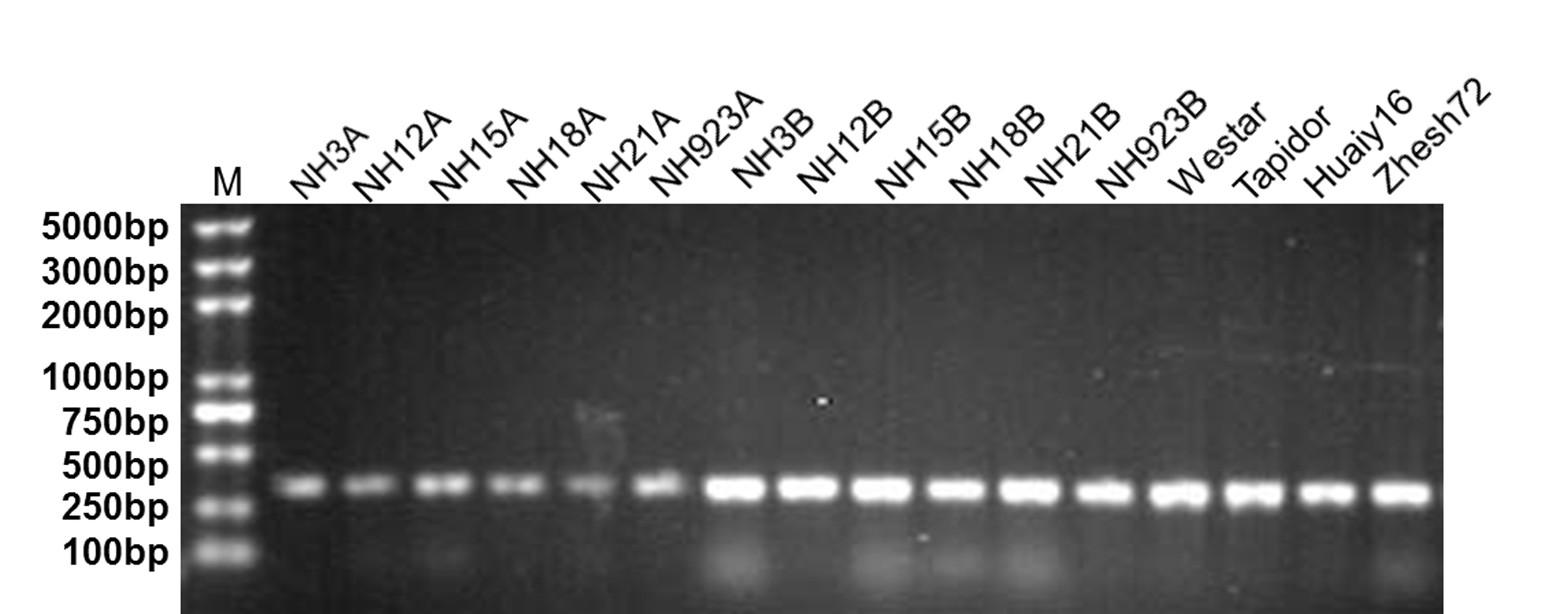

Supplement: Figure S1 — PCR analysis of a panel of male fertile and pol-CMS cultivars using P1. Analysis was based on the nap-specific primer pair P1, which targeted orf117b, but amplification occurred in both nap- and pol-CMS lines. Huaiy16: Huaiyin16; Zhesh72: Zheshuang72; M: DNA ladder. (TIF) [file pone.0017662.s001.tif]

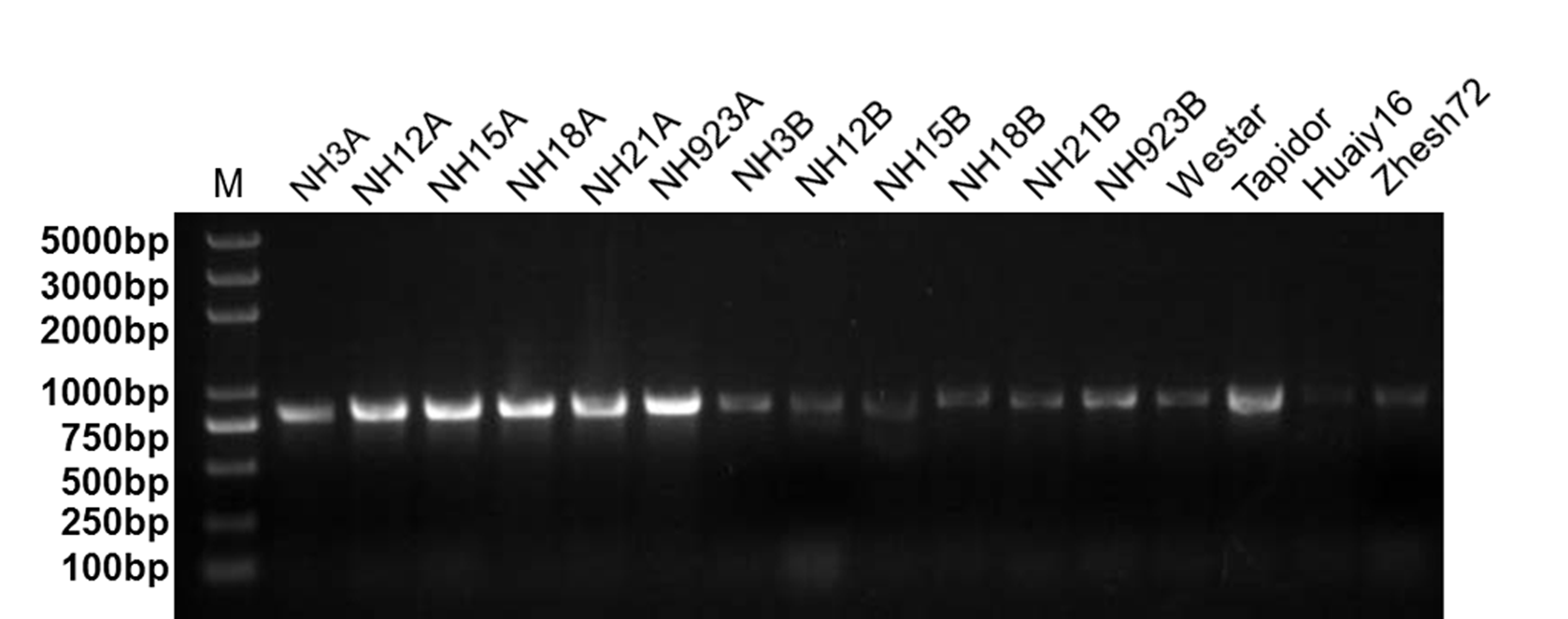

Supplement: Figure S2 — PCR analysis of a panel of male fertile and pol-CMS cultivars using P5. Analysis was based on the pol-specific primer pair P5. Amplification of the target fragment occurred in both nap- and pol-CMS lines. Huaiy16: Huaiyin16; Zhesh72: Zheshuang72; M: DNA ladder. (TIF) [file pone.0017662.s002.tif]

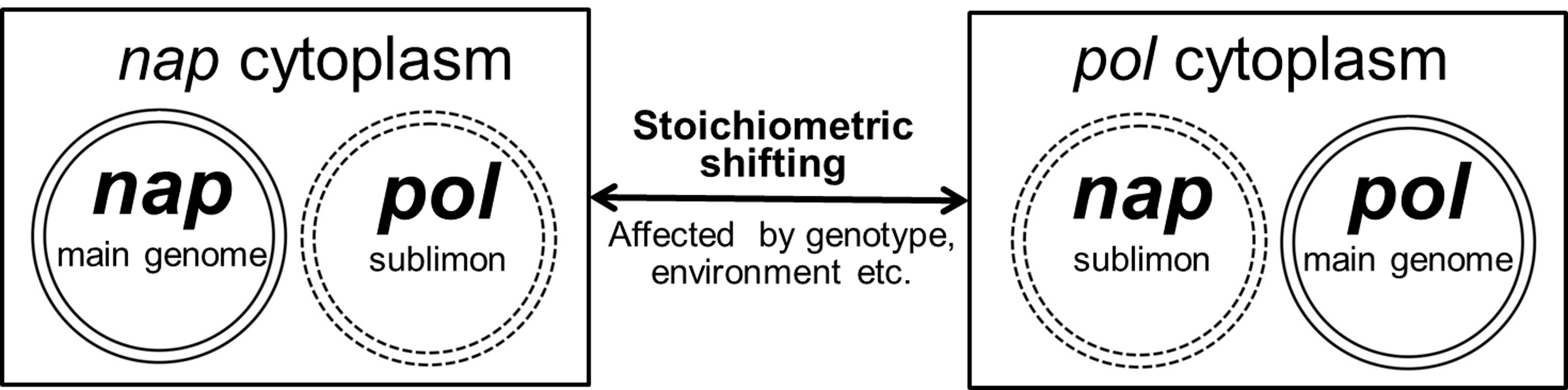

Supplement: Figure S3 — The coexistence and stoichiometric shifting of the nap and pol mitotypes in B. napus. Nap and pol mitotypes coexisted in B. napus, but the relative content of the two mitotypes was different in the two cytoplasm types. In the nap cytotype, nap is the prevalent mitotype and pol is present as a sublimon. In the pol cytotype, pol is the prevalent mitotype and nap is present as a sublimon. Under certain conditions, including nuclear genotypes and environmental stress, sublimons may be amplified and accumulate to take over the role of the main genome. (TIF) [file pone.0017662.s003.tif]
