# Supplementary material for: Substoichiometrically Different Mitotypes Coexist in Mitochondrial Genomes of Brassica napus L
Source: PLoS One. 2011 Mar 10;6(3):e17662. doi: 10.1371/journal.pone.0017662 (PMC3053379; doi:10.1371/journal.pone.0017662)
Supplement: Table S1 — Different ORFs between nap and pol mitotypes. (DOC) [file pone.0017662.s004.doc]

**Table S1. Different ORFs between *nap* and *pol* mitotypes.**

| ***Pol* mitotype** | ***Nap* mitotype** | **Similarity of nucleotide sequence** | **Similarity of amino acid sequence** | | **Notes** | |
| --- | --- | --- | --- | --- | --- | --- |
| *orf118* | *orf124* | 91% | | 66% | |  |
| *orf293* | *orf286* | 96% | | 97% | |  |
| *orf113a’* | *orf113a* | 99% | | 99% | | 1 bp substitution |
| *orf195’* | *orf195* | 99% | | 99% | | 1 bp substitution |
| *orf265* | *orf261* | 74% | | 62% | | 467 bp shared by both mitotypes |
| *orf305* | *orf322* | 94% | | 94% | | 51 bp missing in *pol* mitotype |
| *Ψorf106a* | *orf106a* | 99% | |  | | Disrupted by 2 bp insert in *pol* mitotype |
| *-* | *orf188* |  | |  | | 90 bp of *orf188* found in *pol* mitotype |
| *-* | *orf117b* |  | |  | | Unique to *nap* mitotype |
| *orf122* | *-* |  | |  | | Unique to *pol* mitotype |
| *orf132* | *-* |  | |  | | Unique to *pol* mitotype |

- indicates the ORF is absent in the mitotype.
