# Supplementary material for: Substoichiometrically Different Mitotypes Coexist in Mitochondrial Genomes of Brassica napus L
Source: PLoS One. 2011 Mar 10;6(3):e17662. doi: 10.1371/journal.pone.0017662 (PMC3053379; doi:10.1371/journal.pone.0017662)
Supplement: Table S2 — PCR primer sequences. (DOC) [file pone.0017662.s005.doc]

**Table S2.** PCR primer sequences.

| Name | Primer sequences | Ta(℃) | Product length (bp) | |
| --- | --- | --- | --- | --- |
|  |  |  | *Pol* | *Nap* |
| P1 | F: 5’-TGGGTATGGGTCAGGACAGT-3’  R: 5’-GGTGGGCATCCAAGACAAG-3’ | 58 | — | 335 |
| P2 | F: 5’-GTACAAGTAAGTTCGGGGTCT-3’  R: 5’-TGGTGATCCAAGCTCCTG-3’ | 53 | — | 391 |
| P3 | F: 5’-GTAGTTCAGATTCAAGTCGGT-3’  R: 5’-GCGTACATGTCATTATTTCG-3’ | 51 | 286 | — |
| P4 | F: 5’-CTCACGCACAATCCACAAGA-3’  R: 5’-CAAAAGCATGGGAGAAAACC-3’ | 57 | 1425 | — |
| P5 | F: 5’-CATCCCTCGCTCTGGGTGAC-3’  R: 5’-TGGGCTTTCTTGGATTCGTTT-3’ | 60 | 821 | — |
| P6 | F: 5’-GAATCCAAGAAAGCCCAAAA-3’  R: 5’-GGAAAGAATGCTTAGCGACAC-3’ | 57 | 1528 | — |
| P7 | F: 5’-GAAGCAAGCCCGCCTGGTGT-3’  R: 5’-GAGTAGGGAGTCAATCTGAAAGG-3’ | 57 | 1524 | — |
| P8 | F: 5’-TATTGAGGTGTTTATCGGGTTC-3’  R: 5’-TGTTCCTTTACCAGGTTCAGC-3’ | 57 | 1531 | — |
| P9 | F: 5’-AAACTGAACGAAAGCGGTAG-3’  R: 5’-ACAGACGGTATCTCCCCATG-3’ | 57 | 797 | — |
| P10 | F1: 5’-GGATGAAACTGAATGACCAAA-3’  F2: 5’-TTCTGTTGGATGAAACCTTACTT-3’  R: 5’-CAGGAGAAGTCGCTTATGGAA-3’ | 57 | 841 | 226 |
| P11 | F: 5’-GGAATGTCATCATAAGGGTGTT-3’  R: 5’-AATCCAGTGAGTAGAAGCGGTA-3’ | 55 | 767 | — |

Ta represents annealing temperature.
