# Supplementary material for: Substoichiometrically Different Mitotypes Coexist in Mitochondrial Genomes of Brassica napus L
Source: PLoS One. 2011 Mar 10;6(3):e17662. doi: 10.1371/journal.pone.0017662 (PMC3053379; doi:10.1371/journal.pone.0017662)
Supplement: Table S3 — Primers and probes for TaqMan qPCR. (DOC) [file pone.0017662.s006.doc]

**Table S3**. Primers and probes for TaqMan qPCR.

| **Primer name** | **Primer and probe sequences** | **Ta(℃)** | **Product length (bp)** |
| --- | --- | --- | --- |
| ***orf222*** | F: 5’-GGTGGAAAAGATCGTACAAGTAAGT-3’  R: 5’-CCGACGCCGAACTGAAAT-3’  P: 5’-fam-CGCTATTTCCTCATGTTCGTGGTCCCA-tamra-3’ | 58.5 | 173 |
| ***orf224*** | F: 5’-ATCGTAGTTCAGATTCAAGTCGGT-3’  R: 5’-CGACGCCGAAATGACAAAT-3’  P: 5’-fam-TGGCCGCCCATTATTTTATCATTTTCG-tamra-3’ | 58.5 | 168 |

Ta represents annealing temperature.
